# Basosquamous Carcinoma: A Rare Entity With an Atypical Presentation

**DOI:** 10.7759/cureus.66320

**Published:** 2024-08-06

**Authors:** Madiha Eljazouly

**Affiliations:** 1 Dermatology Unit, Cheikh Khalifa International University Hospital, Mohammed VI University of Health Sciences, Casablanca, MAR

**Keywords:** dermoscopy, skin tumor, squamous cell carcinoma, keratinizing tumor, basal cell carcinoma, basosquamous carcinoma

## Abstract

Basosquamous carcinoma (BSC) is a rare entity of basal cell carcinomas. It is described as being nosologically at the border between a squamous cell carcinoma and a basal cell carcinoma, thus sharing characteristics of both entities. The frequency of this pathology remains low with a few cases reported in the literature. We report the observation of a basosquamous carcinoma with a particular topography on the pulp of the left fifth finger. A histological examination confirmed the diagnosis. Locoregional and general extension studies were negative. Management consisted of surgical resection.

## Introduction

Basosquamous carcinoma (BSC) is an uncommon, controversial entity of non-melanoma skin cancer. It is described as nosological, at the border between squamous (SCC) and basal cell carcinoma (BCC). Some authors considered BSC merely a variant of BCC and others have suggested that it represents a collision of separate primary BCC and SCC [1,2]. Thus, it presents a real diagnostic challenge because of its variable clinical and histologic features. This current controversy extends to classification and involves pathogenesis and its management.

BSC is a rare and aggressive cutaneous neoplasm. The frequency of this pathology remains low with a few cases reported in the literature. It is usually located in the head and neck or other sun-exposed areas with rare cases in the dorsum of the hand [3,4]. In a study published by EJ van Zuuren et al., 2990 BCCs were registered, with 110 located on the upper extremities. Among these tumors, 11 patients were identified as having a BCC on the dorsum of the hand, two of which were diagnosed as BSC [4]. The diagnosis of BSC remains primarily histological although dermoscopic evaluation can provide important information. Here, we present a case of basosquamous carcinoma with an unusual location on the palm.

## Case presentation

**Figure 1:**
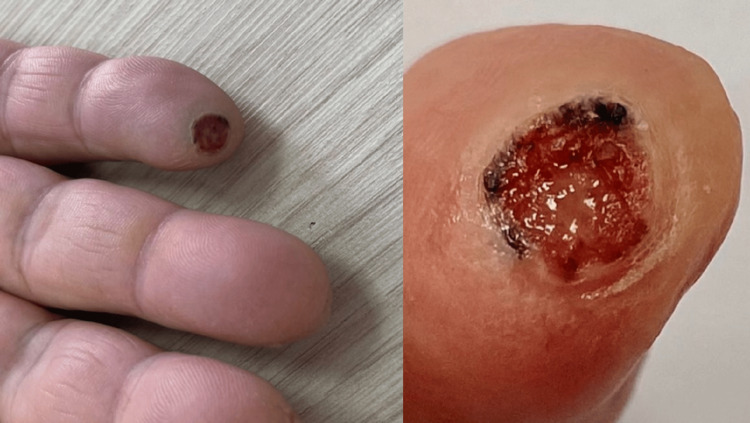
Nodular ulcerated lesion

A 68-year-old woman, treated for type II diabetes and dyslipidemia, presented to the dermatology department for a nonpainful, nonpruritic ulcerated skin lesion, evolving for two years, with no spontaneous amelioration. The patient reported the application of topical antibiotics. The clinical examination revealed a well-limited circumscribed ulceration, measuring 1 cm x 1 cm, located in the left fifth finger pulp, with a clean bottom, bleeding on contact, surrounded by an inflammatory halo, made of a few translucent pearled vesicles, as well as a peripheral blackish pigmentation (Figure 1). The mucocutaneous examination was unremarkable. The examination of lymph nodes was normal, as well as the rest of the organs.

**Figure 2:**
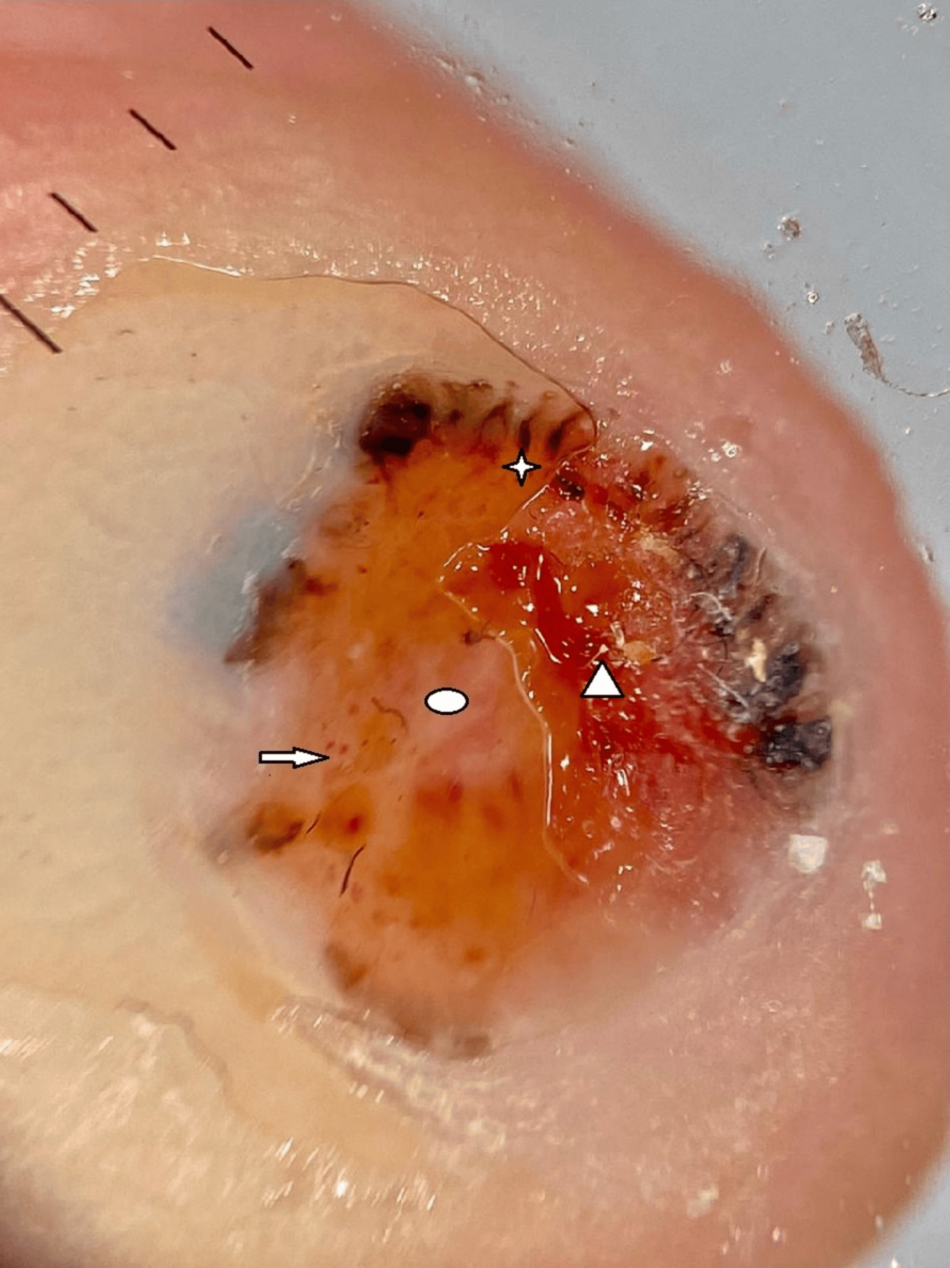
Dermoscopy shows a central crust covering an ulceration and a white scale (triangle). On the periphery are brown dots (star), a polymorphic vascular pattern (arrow), and white-pinkish structureless areas (circle).

Dermoscopy revealed dotted vessels, hairpin vessels, ovoid nests, digitiform structures at the periphery, ulceration, and white structureless areas (Figure 2).

**Figure 3:**
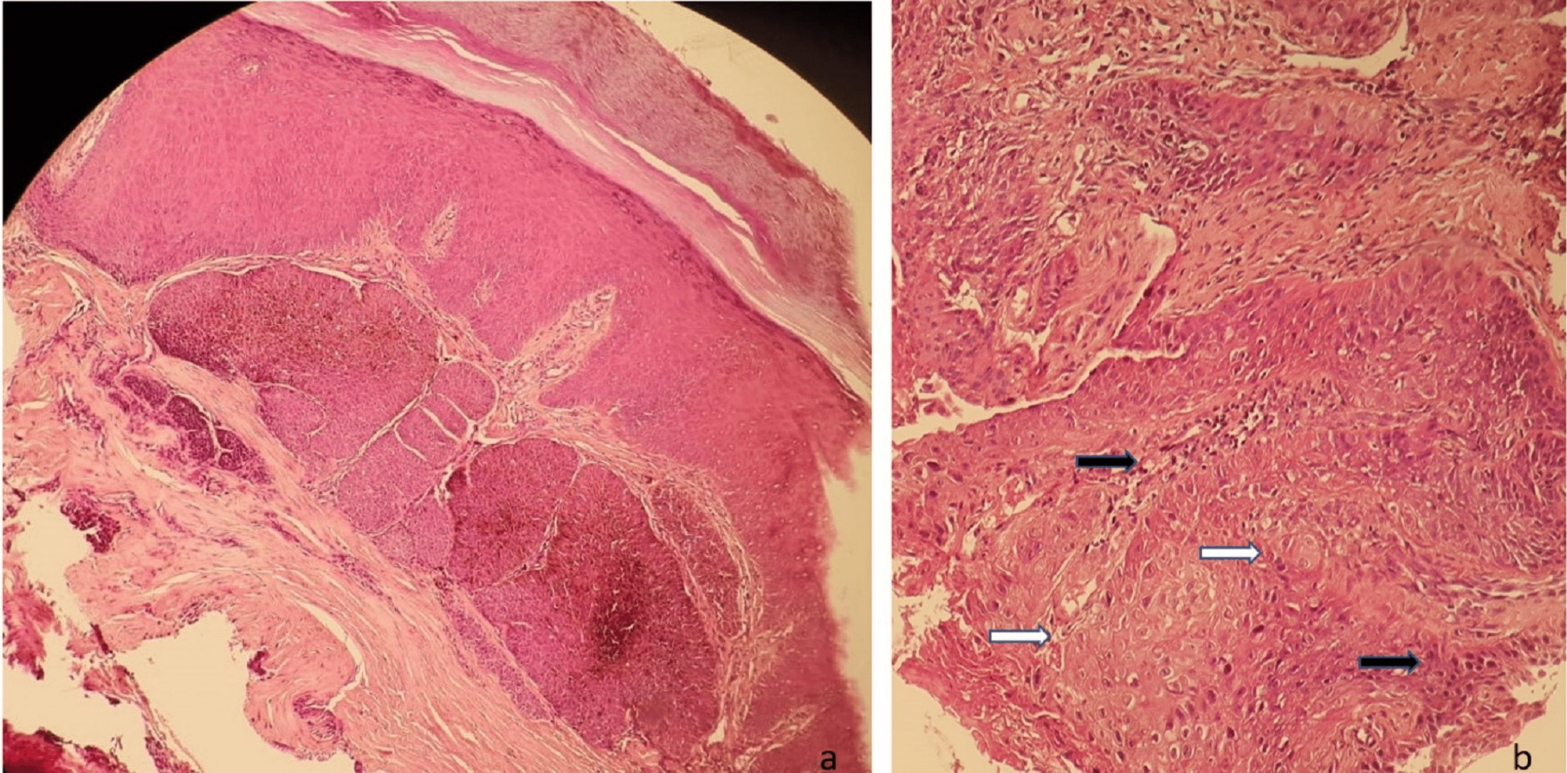
Histologic characteristics of BSC Aggregations with peripheral palisading of basaloid cells (black arrows) are accompanied by atypical keratinocytes in the center with a transition zone and squamous differentiation (white arrows). Hematoxylin-eosin stain; a: x10; b: x20 BSC: basosquamous carcinoma

The histological study revealed an infiltrative growth of basaloid cells with large cytoplasm and pale nuclei besides aggregates of squamous cells, leading to the diagnosis of basosquamous carcinoma (Figures 3a, 3b). Management consisted of surgical resection with 5 mm margins. Locoregional and general extension studies were negative. The patient has remained free of recurrence for 24 months.

## Discussion

BSC was first described in 1894 by Beadles as a collision of two co-existing carcinomas, then as an intermixed tumor in 1910 by MacCormac [2,5]. The WHO (World Health Organization) defines it as an aggressive variant of BCC possessing elements of cell differentiation specific to SCC [6]. However, most authors agree to classify this entity as a carcinoma in its own right, regarding clinical and dermoscopic presentations and for its specific management. The incidence of basosquamous carcinoma is estimated to be between 1.2% and 2.7% of all cutaneous carcinomas, with most lesions occurring in the head and neck region. Risk factors include Fitzpatrick skin types 1-2, ultraviolet radiation, advanced age, male gender, and tobacco use [2,7]. Clinically, it is a rounded nodular lesion, chronically evolving toward ulceration, located mainly in the photo-exposed areas, with few cases reported on the trunk and extremities [8]. BSC has no characteristic clinical presentation so it can provide a real differential diagnosis with several diseases: leishmaniasis, tumors (achromic melanoma, squamous cell carcinoma, Bowen’s disease, keratoacanthoma), or even pyogenic granuloma.

Dermoscopy remains an interesting tool for directing the diagnosis. It is based on a combination of SCC and BCC patterns. However, it should be noted that the dermoscopic signs of BSC have been little studied and are not very specific.

The dermoscopic aspects of findings are the presence of a keratin mass, blood crusts, surface scale, ulceration white structures made of circles and clods, blue-gray areas, radial lines, and arborescent vessels [5]. A study published by Akay et al. demonstrated the value of dermoscopy in diagnostic orientation, highlighting the correlation with anatomopathological study. Cases were classified as dominant BCC, intermediate, and dominant SCC. Keratin masses and hemorrhagic spots on keratin masses were observed in all SCC-dominant types, whereas, white structures (white circles, white clods, white lines, and white structureless areas) were identified by dermoscopy in the majority of the lesions. Additionally, the blue-gray areas characteristic of BCC were frequent in the BCC-dominant type. Furthermore, the vascular structures were observed in all lesions, predominating branched and serpentine vessels [9].

However, the diagnosis remains histological, after eliminating a collision of BCC and SCC or a keratinizing BCC. It’s based on the association of at least one characteristic of each type of carcinoma. Some authors consider that BSC begins initially a basal cell carcinoma with genetic alterations that lead to squamous differentiation. Currently, the most commonly adopted histological definition of BSC is an infiltrative growth of basoloid cells with large cytoplasm and uniform nuclei besides the squamous cell component, with a transition zone made of pluripotential basal cells and a fibroblast-rich, collagenized stroma. Immunostaining, particularly Ber-EP4, is important for completing the histological study in cases of doubt; it is a marker for basaloid differentiation and characterizes the transition zone [7,10,11]. However, in our case, we did not carry out Ber-EP4 immunostaining, as the diagnosis was clearly established through histopathological examination.

To date, there is no consensus or standardized uniform medical approach regarding managing this type of carcinoma [12]. Mohs micrographic surgery is the first-line treatment for a single lesion with negative extension. In our case, Mohs surgery was not performed due to its unavailability at our facility; instead, we opted for standard excision, which was deemed appropriate given the characteristics of the lesion. The sentinel lymph node method is indicated for lesions larger than 2 cm or invasion of the peri-nervous sheath [13,14]. Chemotherapy, radiotherapy, and immunotherapy remain indicated in case of incomplete margins, lymph node metastasis, or distant metastasis. Despite its usually nonspecific and benign clinical appearance, BSC is considered an aggressive tumor with high metastatic potential, estimated at 5-10% and a recurrence rate of up to 12-51% for surgical excision and 4% for Mohs micrographic surgery [2,5,15]. The particularity of our observation is the occurrence of this rare carcinoma in a middle-aged patient, with a dark phototype, on an uncommon and non-photo-exposed area.

## Conclusions

The classification, pathogenesis, histological morphology, biological behavior, prognosis, and management of BSC are subjects of ongoing controversy and debate. Although BSC is rare, it is crucial to recognize due to its metastatic potential and high recurrence rate. In this case, the tumor did not recur following standard excision; however, Mohs micrographic surgery is preferable when available, and immunostaining should be considered in cases of doubt. This case underscores the importance of considering BSC in differential diagnoses and highlights the need for early detection and appropriate treatment to improve patient outcomes.
